# Educating speech-language pathologists working in early intervention on environmental health

**DOI:** 10.1186/s12909-018-1266-3

**Published:** 2018-07-03

**Authors:** Emily Zimmerman, Catherine Borkowski, Stephanie Clark, Phil Brown

**Affiliations:** 1 Department of Communication Sciences & Disorders, Northeastern University, 360 Huntington Ave, Boston, MA 02115 USA; 2 Department of Sociology, Northeastern University, 360 Huntington Ave, Boston, MA 02115 USA; 3 Department of Health Sciences, Northeastern University, 360 Huntington Ave, Boston, MA 02115 USA

**Keywords:** Early intervention, Speech-language pathology, Environmental health, Education

## Abstract

**Background:**

The goals of this study were (1) to determine early intervention (EI) Speech-Language Pathologists’ (SLPs) level of training and knowledge on environmental toxicants and their effect on infant and child development; and (2) to examine the effectiveness of a continuing education (CE) event designed to enhance the knowledge of EI SLPs on environmental toxicants and their effects on child development.

**Methods:**

A survey was launched via Qualtrics and posted on the American Speech-Language Hearing Association’s Early Intervention Community page to assess environmental health knowledge of SLPs. Results from this survey were used to create an environmental health CE event targeted towards EI SLPs. Attendees were given a pre- and post-test to assess the effectiveness of our program.

**Results:**

One hundred and fifty-eight participants completed the online survey and a majority (61%, *n* = 97) of participants reported some level of dissatisfaction with their previous training in regards to environmental exposures. Fifty-six percent (*n* = 89) of the participants also reported feeling unprepared to be a health advocate regarding environmental exposure concerns within their community. Forty-eight people (26 SLPs and 22 SLP master’s students) attended the CE event. Paired t-tests revealed significant improvements from the pre- to the post- test results among all attendees.

**Conclusions:**

These findings suggest that SLPs who work in EI feel undertrained and unprepared to advocate for environmental health to the families they serve. This study reveals that CE is one way by which to increase the knowledge base of SLPs on environmental health.

**Electronic supplementary material:**

The online version of this article (10.1186/s12909-018-1266-3) contains supplementary material, which is available to authorized users.

## Background

Environmental health factors play a large role in children’s health due in part to the fact that children are more susceptible to these factors than adults [1]. Childhood is a period of rapid growth and development characterized by changes in organ system functioning, metabolic capabilities, physical size, and child behaviors (e.g., hand-to-mouth and hand-to-toy behaviors), which can all be modified by toxicant exposures. Put simply, children are at increased risk because they breathe more air, drink more water, eat more food per kilogram than adults, and play closer to the ground where many contaminants are found. Childhood exposures begin prenatally and extend through early adolescence. Different developmental phases, known as “windows of vulnerability,” can result in different susceptibilities to the effects of toxicants or manufactured environmental toxicants (METs) [2, 3]. Recently, the public media and the scientific community alike have begun to examine the connections between exposures to METs in relation to the unexplained rise in complex disorders with multifactorial origins, such as Autism Spectrum Disorders, developmental delays, attention deficit hyperactivity disorders (ADHD), asthma, learning disabilities, cancer, endocrine pathology, and autoimmune disorders [4, 5].

The economic and neurological consequences of environmental exposures are high. The annual costs of environmentally attributable diseases such as lead poisoning, asthma, childhood cancer, and neurobehavioral disorders is about $54.9 billion across American children [5]. These financial costs fall not only on the families of affected children, but also on government programs, such as early intervention and public schooling. Additionally, the neurodevelopmental and disease costs can be devastating for both children and their families. According to the World Health Organization (WHO), more than 30% of the global burden of disease evident in children is due to environmental factors [6]. Lead, mercury, polychlorinated biphenyls (PCBs), flame-retardants, and pesticides have all been shown to result in intellectual deficits in children [4, 7]. In fact, Bellinger compared common children’s health problems (birth defects, preterm birth, ADHD, Autism, brain injuries) to lead, organophosphate pesticides, and methylmercury [8] and found that the three environmental exposures together would decrease population-wide children’s IQ by 40 million points compared to the 34 million IQ points for preterm birth, 17 million IQ points for ADHD, and 7 million IQ points for Autism. This study suggests that parents and health care providers should be aware of common environmental exposures and how to prevent them, especially given their relationship to developmental delays. A review by Dzwilewski and Schantz suggests that these overall reductions in intellectual function likely hinder language development as well; therefore, it is important for clinicians and researchers in communication sciences and disorders to be aware of these findings [9].

While pediatricians and other medical professionals can help educate families on environmental health, early ntervention (EI) Speech Language Pathologists (SLPs) can play a major role, as they often spend their sessions in the family’s home or childcare facility, where the child could potentially be exposed to dangerous toxicants on a daily basis. Additionally, the environmental toxicants could account for some of the origin of the delays that are evident in the child that they are assessing or treating. Therefore, SLPs are uniquely suited to both educate caregivers on the importance of environmental health and to treat some of the neurodevelopmental outcomes that are the result of exposures to various toxicants; however, it remains unclear if and to what extend SLPs are trained in or knowledgeable about environmental health issues. Therefore, this study had two aims: 1) to determine EI SLPs’ training and knowledge on environmental toxicants and their effect on infant and child development (Phase 1); and 2) to examine the effectiveness of a continuing education event designed to enhance the knowledge of SLPs working in EI on environmental toxicants and their effects on infant and child development (Phase 2).

## Methods

### Methods phase 1: EI SLPs’ knowledge of environmental health

A Qualtrics® survey, software version 2016 of Qualtrics (Provo, Utah), tailored to SLPs working in EI was designed to assess overall knowledge of environmental health. There were fourteen questions in our survey. The first three questions asked participants about their SLP job characteristics. Questions 4–13 focused on environmental health training and knowledge. Question 14 asked what specific areas of environmental health issues EI SLPs would like to learn more about. See Additional file 1: Qualtrics Survey Questions for the complete survey. Once the survey page was designed, an initial review of the survey was completed internally in the lab, where the appropriateness of each question was assessed. Next, the link was sent to colleagues to ensure that the survey was functional and fully operational without any system errors and to confirm that the data could be accurately exported. After preliminary testing, the survey link was posted on the American Speech-Language-Hearing Association’s (ASHA) Early Intervention Community (2.5 k members) webpage for approximately one month. The survey was designed to be brief (5 min) and targeted to attract the maximum number of respondents. Participants in the online survey were notified that the participation was optional and that the results would be published; therefore, completion of the survey indicated implied consent.

### Methods phase 2: SLP continuing education event

**Fig. 1:**
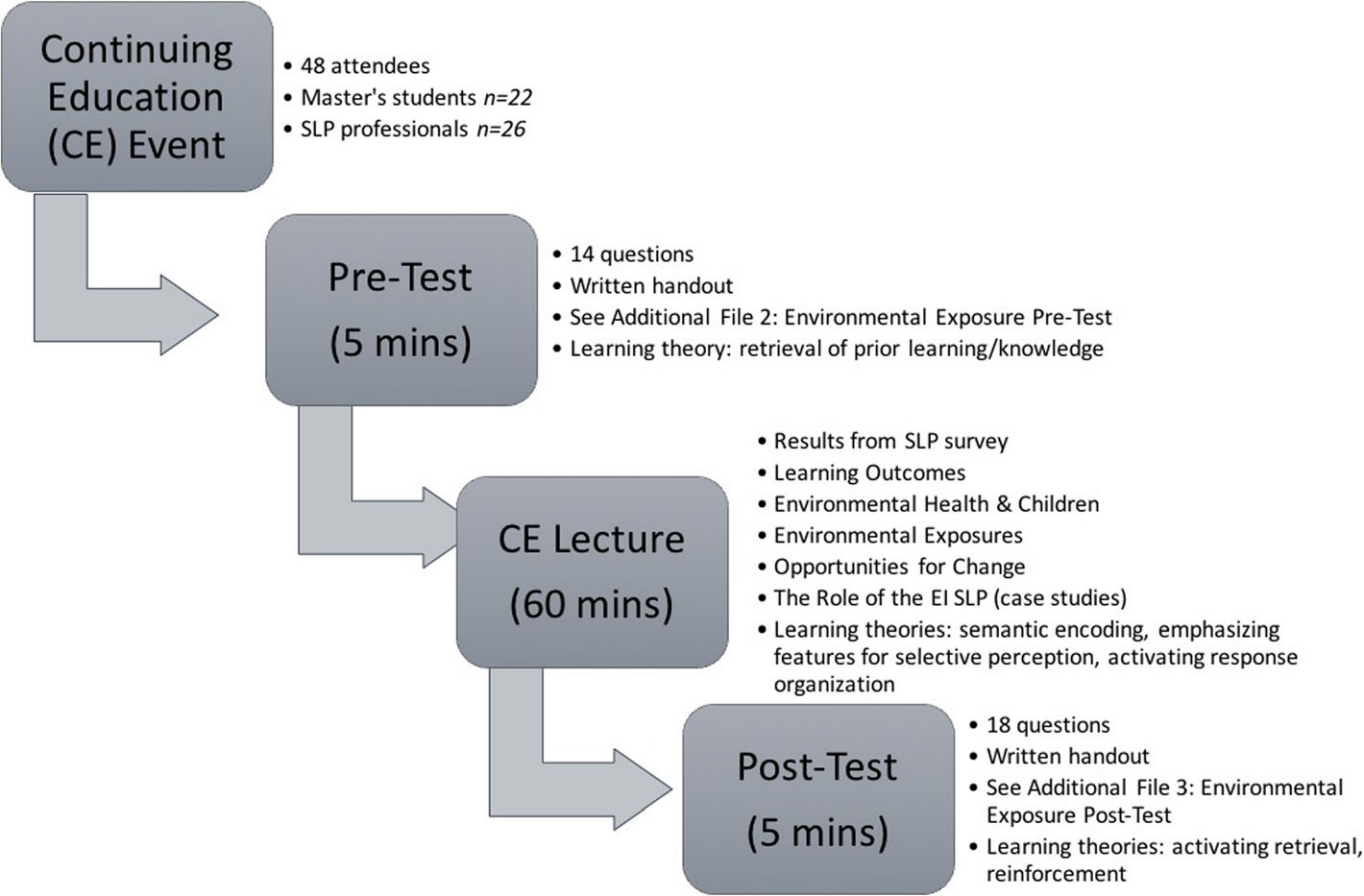
Flow Chart describing the CE event and learning theories associated with each part of the event

Based on the survey results from Phase 1, a continuing education (CE) event at Northeast of the United States, entitled, “An Early Intervention Speech-Language Pathologist CE Event” was created, see Fig. 1. EI SLPs in the greater Boston area were invited to the event and master’s students in our Communication Sciences & Disorders program were encouraged to attend. The two-hour event featured the following one-hour presentations:“Environmental Health Exposures: What Early Intervention Speech-Language Pathologists Need to Know” and “Developments in Play for Infants and Toddlers with Delay: Implications for Intervention”—both topics of interest to SLPs working in EI. The learning outcomes of this event specific to the environmental health presentation were as follows:

1. To determine the effect of common environmental exposures on infant and child development.
2. To examine how environmental exposures affect speech and language development.
3. To determine the role of early intervention SLPs in relation to environmental health in homes.

In order to examine the efficacy of the event, an environmental health pre- and post- test was created (see Additional file 2: Environmental Exposure Pre-Test and Additional file 3: Environmental Exposure Post-Test, respectively). Participants in the CE event were told that the participation in the pre- and post- testing was optional and that their results would be published; therefore, completion of these documents implied consent. The pre- and post- test consisted of the same 14 questions designed to ask specific questions about environmental health. The pre-test was taken prior to the beginning of the presentation and was designed to assess the participant’s prior environmental health knowledge. The post-test was distributed and taken in the last five minutes of the presentation and was designed to activate retrieval of newly acquired environmental health information and to determine if the learner outcomes were attained. Participants also completed a 7-question program evaluation for each presentation, which was scored using a Likert scale of 1–5 (5 being the highest score). The 26 SLPs who attended the event were all female and on average 36 years old (± 10.83), had 12.20 years (± 10.43) of experience working as an SLP, and 5.12 years (± 6.62) working in EI. The 22 SLP master’s students who attended the event were on average 23 years old (± 3.82) and 21 were female.

## Results

### Results phase 1: EI SLPs knowledge of environmental health

One hundred and fifty-eight participants completed the survey. A majority (60%; *n* = 95) reported that they had not received specific training regarding the effect of environmental exposures on child development. Regardless of training, 78% (*n* = 124) of participants reported that the role of environmental health on child development is very important; however, only 24% (*n* = 38) reported *always* considering environmental health factors during EI. The SLPs surveyed reported talking to parents/caregivers about diet/food choices, housing/home environment, school/childcare environment, and drugs. Participants were asked “are there any specific environmental issues (that you are aware of) that impact the population that you treat? (You may select more than one)” and they responded as follows: “air pollution (including secondhand smoke exposure)” (26%, *n* = 42), “lead exposure” (24%, *n* = 38), “pesticide exposure” (8%, *n* = 13), “drug/alcohol exposure” (4%, *n* = 7), and “diet” (3%, *n* = 5). Seventeen percent (*n* = 27) of participants reported “none” and 35% (*n* = 56) reported other issues. A majority of participants (61%, *n* = 97) reported some level of dissatisfaction with their level of training in regards to environmental exposures and their effect on child development. In fact, 56% (*n* = 89) reported feeling unprepared to be a health advocate about environmental exposure concerns within their community. When asked what specific areas of environmental health they would like to learn more about, participants reported poor nutrition, pesticides, drug use, household cleaners and chemicals, air pollution, and genetically modified organisms (GMOs).

### Results phase 2: SLP continuing education event

**Fig. 2:**
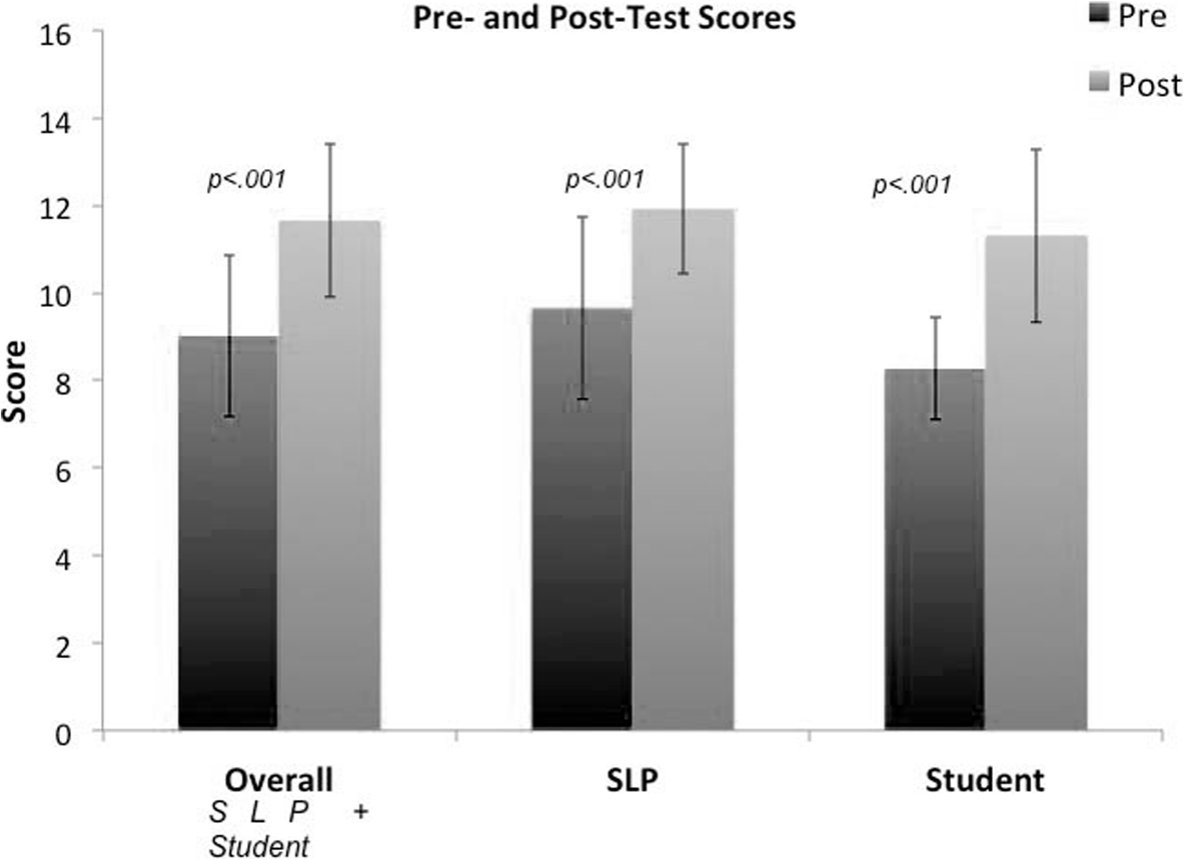
The pre- (dark gray) and post- (light gray) test results from the CE program for SLPs and master’s students

Twenty-six SLPs and 22 SLP master’s students attended the CE event (48 total). Paired t-tests revealed there to be a significant difference in the pre- and post- test results in all attendees (SLPs + Students) [t(47) = − 8.45*, p < .001*], in SLPs only [t(25) = − 5.48, *p < .001*], and students only [t(21) = − 6.58, *p < .001*], see Fig. 2. This demonstrates that the aforementioned learning outcomes were also met, given the statistically significant nature of these data.

**Fig. 3:**
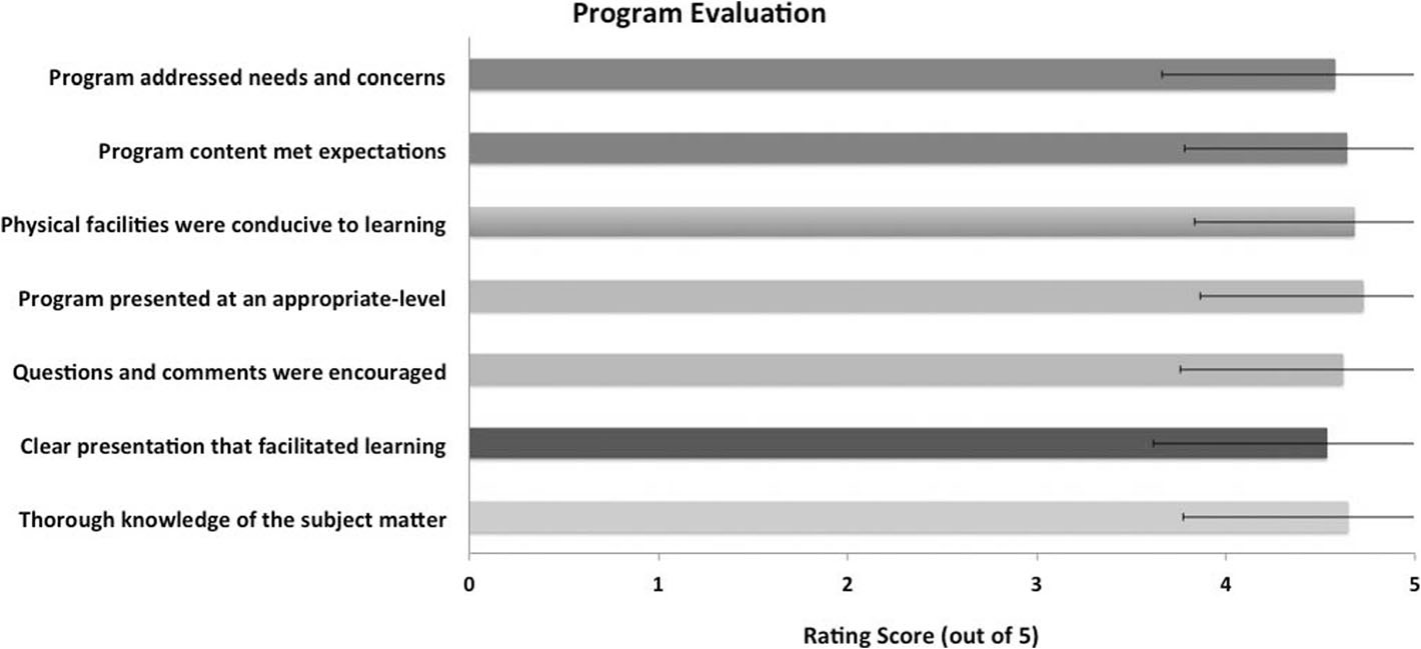
SLPs’ evaluation of CE event on environmental health across seven categories, using a Likert scale rating with five being the highest possible score

The program evaluation averages on a 5-point Likert Scale (with 5 being the highest; Fig. 3) were as follows: thorough knowledge of the subject matter 4.64 (± .86), clear presentation that facilitated learning 4.53 (± .91), questions and comments were encouraged 4.61 (± .86), program presented at an appropriate level 4.72 (± .86), physical facilities were conducive to learning 4.68 (± .84), program content met expectations 4.63 (± .85), and program addressed needs and concerns 4.57 (± .91). When asked what was the most helpful aspect of the presentation, 18 reported the case studies, 8 reported the real life environmental health examples, and 7 reported information on toxicants.

## Discussion

This is the first study to examine the knowledge of EI SLPs on environmental health. The outcomes from Phase 1 of the study were extremely informative in that they showed that SLPs reported having very little education on environmental toxicants and being dissatisfied with the amount of training they have received. This information is disconcerting, especially considering that EI SLPs have the unique opportunity to go into client’s homes and interact with their families, giving them the ability to act not only as clinicians, but also as educators. Furthermore, the survey indicated an interest on the part of SLPs to receive more education on this topic, which led to the creation of the environmental health CE event in Phase 2.

The pre- and post- surveys from the CE event revealed that all participants significantly increased their knowledge of environmental health. It is important to note that the pre- and post-tests were designed to be challenging for the participants and looked at very specific details regarding environmental toxicants and their effect on infant and child development. Therefore, the fact that all participants improved on this measure indicates that the event was effective at educating SLPs and students alike on both broad and specific environmental health issues. It is important to note that, we only sampled short-term learning outcomes and did not follow our cohort over time. We speculated that while the short-term learning may disappear, we hoped that our CE event enhanced an interest in areas of environmental health to spur SLPs and students to further their learning and engagement in this area. Subsequent studies should examine the long-term effects of these types of CE events as well.

The program evaluation scores of the CE event were high. Participants reported that the case studies and environmental health examples were very helpful components of the presentation. Case study presentations led to a discussion regarding the role of the SLPs in environmental health and leveraged the learning theories of activating response organization as well as emphasizing features for selective perception, which have been shown to improve learning gains [10, 11]. Within ASHA’s statement on the role of an EI SLP, there are four guiding principles that reflect the current consensus on best practices for providing effective EI services [12]. Specifically, services should be (a) family-centered and culturally responsive; (b) developmentally supportive and promote children’s participation in their natural environments; (c) comprehensive, coordinated, and team-based; and (d) based on the highest quality internal and external evidence that is available [12]. Environmental health knowledge clearly fits within all of these guiding principles.

Taken together, the results from Phases 1 and 2 indicated the need for further education of SLPs working in EI, and other areas of practice, regarding environmental toxicants and their effects. Though we began by surveying EI SLPs, as they are the most likely provide services in clients’ homes, the results from the Phase 1 survey revealed that all SLPs would benefit from more environmental health training. There are several ways by which to disseminate environmental health knowledge to SLP clinicians and students, including: 1) offering a course or lecture series in graduate school, 2) hosting continuing education events, and 3) providing practicing SLPs with resources. We asked 13 SLPs who attended our poster session presenting these data at the 2016 ASHA National Conference about the best way to disseminate this knowledge. Sixty-nine percent of participants reported preferring dissemination through continuing education, 23% of preferred teaching this subject in graduate school, and 8% reported that SLPs could seek out this information independently with no formal mechanism necessary.

Guidelines should be developed to specify what knowledge and training SLPs and other medical professionals should have in regards to environmental health. These should include the following topics: common environmental exposures in the home/school/childcare environment, food choices, air pollution, and other chemical exposure mixtures. SLPs should also be prepared to discuss environmental topics in the news. This might include, for example, lead levels in water and/or where families can have their child tested for lead exposures. University education programs and the accrediting agency would need to take responsibility for providing such education, as we would not expect SLPs to seek it individually.

There were several limitations to this study. While Phase 1 was circulated nationally, it only accounted for 6.30% of all SLPs accredited by ASHA. Thus, these results may not generalize to all SLPs and results should be taken with caution. In addition, 158 is a relatively small sample size, especially when considering that the ASHA community we posted the survey on has 2500 members. Future studies may collect a larger sample size, perhaps including SLPs practicing in other areas of the field (i.e., geriatric home health).

## Conclusion

Taken together, results from this study suggest that SLPs who work in EI feel undertrained and unprepared to advocate for environmental health to the families they serve. Our study reveals that CE is one way by which to increase the knowledge base of SLPs on environmental health. The next steps towards educating SLPs on environmental health will be to expand our current CE event to include SLPs working in various settings (e.g., home health, rehab, hospitals) and to other medical professionals (physicians, nurses, occupational therapist, physical therapists, etc.,) across the country and internationally.

## Additional files

Additional file 1: Qualtrics Survey Questions. The fourteen questions in our Qualtrics survey were tailored to speech-language pathologists working in early intervention. The survey assessed overall knowledge of environmental health. Questions ranged from multiple choice to short-answer. (DOCX 19 kb)

Additional file 2: Environmental Exposure Pre-Test*.* The fourteen questions in our environmental exposures pre-test targeted questions about environmental health knowledge. The pre-test was taken prior to the beginning of the CE event and was designed to assess the participant’s prior environmental health knowledge. Data were all multiple choice. (DOCX 24 kb)

Additional file 3: Environmental Exposure Post-Test*.* The fourteen questions in our environmental exposures post-test targeted questions about environmental health knowledge. The post-test was distributed and taken in the last five minutes of the presentation and was designed to activate retrieval of newly acquired environmental health information and to determine if the learner outcomes were attained. Data were all multiple choice. (DOCX 25 kb)

## Abbreviations

ASHA: American Speech-Language-Hearing Association
CE: Continuing education
EI: Early intervention
SLP: Speech-language pathologists
