# Supplementary material for: Educating speech-language pathologists working in early intervention on environmental health
Source: BMC Med Educ. 2018 Jul 3;18:155. doi: 10.1186/s12909-018-1266-3 (PMC6029042; doi:10.1186/s12909-018-1266-3)
Supplement: Supplementary file 1 — Qualtrics Survey Questions. The fourteen questions in our Qualtrics survey were tailored to speech-language pathologists working in early intervention. The survey assessed overall knowledge of environmental health. Questions ranged from multiple choice to short-answer. (DOCX 19 kb) [file 12909_2018_1266_MOESM1_ESM.docx]

**Additional file 1: Qualtrics Survey Questions (Phase 1)**

1. How many years have you been working as a speech-language pathologist?
2. Of those years, how many of those have been spent working in Early Intervention?
3. What state do you currently work in?
4. Have you received any specific training regarding the effect of environmental exposures on child development?
5. Please choose an appropriate response to each item:
   1. The role of environmental health impacts on patients is:
      1. Very important
      2. Somewhat important
      3. Fairly important
      4. Slightly important
      5. Not important at all
6. Please choose an appropriate response to each item:
   1. The amount of control an early intervention SLP has over environmental health hazards is:
      1. No control
      2. Some control
      3. A moderate amount of control
      4. A good amount of control
      5. A great deal of control
7. Please choose an appropriate response to each item:
   1. How often do you consider environmental health factors during early intervention assessment or therapy?
      1. Always
      2. Usually
      3. About half the time
      4. Sometimes
      5. Never
8. How often do you discuss the following exposure sources with patients?
   1. Exposures:
      1. Community/neighborhood
      2. Housing/home
      3. Hobbies
      4. Occupational (adults)
      5. School/child-care (children)
      6. Diet/food
      7. Drugs (including alternative medications and supplements)
      8. Personal habits (i.e. smoking)
   2. Choices:
      1. Always
      2. Most of the time
      3. About half the time
      4. Sometimes
      5. Never
9. To your knowledge, how many children have you seen affected by environmental exposure in the past year in your work?
   1. None
   2. 1 child
   3. 2-4 children
   4. 5-8 children
   5. 9-11 children
   6. 12+ children
10. Are there any specific environmental issues (that you are aware of) that impact the population that you treat? (you may select more than one)
    1. Air pollution
    2. Lead exposure
    3. Contaminated drinking water
    4. Pesticide exposure
    5. None
    6. Other, please specify
11. List up to 4 common exposures that you might advise prospective parents or family with young kids to reduce/avoid:
12. Please choose the appropriate response for each item
    1. Questions:
       1. I am satisfied with my current level of training in environmental exposures as related to child development.
       2. I feel prepared to be a resource to the community if a concern about an environmental exposure health link is raised by the community in which I work.
       3. I feel prepared to be a health advocate in my community on environmental health concerns, especially in regard to child development.
    2. Answer choices:
       1. Strongly disagree
       2. Disagree
       3. Somewhat disagree
       4. Neither agree nor disagree
       5. Somewhat agree
       6. Agree
       7. Strongly agree
13. How confident are you in finding resources about environmental health factors and outcomes, especially in regard to child speech and language development?
    1. Very confident
    2. Moderately confident
    3. Somewhat confident
    4. Not at all confident
14. What specific areas of environmental health issues would you like to learn more about?
