# Supplementary material for: Educating speech-language pathologists working in early intervention on environmental health
Source: BMC Med Educ. 2018 Jul 3;18:155. doi: 10.1186/s12909-018-1266-3 (PMC6029042; doi:10.1186/s12909-018-1266-3)
Supplement: Supplementary file 2 — Environmental Exposure Pre-Test. The fourteen questions in our environmental exposures pre-test targeted questions about environmental health knowledge. The pre-test was taken prior to the beginning of the CE event and was designed to assess the participant’s prior environmental health knowledge. Data were all multiple choice. (DOCX 24 kb) [file 12909_2018_1266_MOESM2_ESM.docx]

**Additional file 2**

ASSIGNED THREE DIGIT NUMBER:_______

**Environmental Exposure Pre-Test:**

1. **Who is more vulnerable to environmental exposure, adults or children?** (circle one)

2. **Choose what is NOT a possible exposure pathway of environmental exposure.**

1. Transplacental
2. Ingestion
3. Inhalation
4. Dermal absorption
5. All of these are potential exposure pathways

3. **Choose 4 health effects of nicotine/secondhand smoke exposure?**

1. Low birth weight
2. Fragile X syndrome
3. Decreased head circumference
4. Ear infections
5. ADHD
6. Cleft palate
7. Autism

4. **Since polychlorinated biphenyls (PCBs) were banned in the US in 1979, are they still an exposure concern for today’s children.** Yes/No (circle one)

5. **Is there a true “safe” level for lead exposure?** Yes/No (circle one)

6. **Each 10-fold increase in DDT level in blood is associated with a 10 to 15 point decrease in mental development.** True/False (circle one)

7. **With long-term exposure, pesticides can affect what body systems?** (choose 4)

1. Endocrine
2. Respiratory
3. Cardiovascular
4. Immune
5. Reproductive
6. Digestive
7. Vision

8. **Where can flame-retardants be found?**

1. Furnishing materials
2. Electronics
3. Plastics
4. Foams
5. All of the above

9. **The “green cleaning” market is highly regulated with many laws and regulations.** True/false (circle one)

10. **What is a major source of BPA (bisphenol A) exposure in children?**

1. Playground equipment
2. Food
3. School supplies (pencils, erasers, etc.)
4. Bubble baths

11. **Children cannot be exposed to air pollution indoors.** True/False (circle one)

12. **Boys are more susceptible** **to behavioral problems associated with increased lead exposure.** Yes/No (circle one)

13. **Are toxic chemicals found in breast milk?** Yes/No (circle one)

14. **Fetuses are protected for environmental toxins *in utero****?*  True/False (circle one)
