# Supplementary material for: Educating speech-language pathologists working in early intervention on environmental health
Source: BMC Med Educ. 2018 Jul 3;18:155. doi: 10.1186/s12909-018-1266-3 (PMC6029042; doi:10.1186/s12909-018-1266-3)
Supplement: Supplementary file 3 — Environmental Exposure Post-Test. The fourteen questions in our environmental exposures post-test targeted questions about environmental health knowledge. The post-test was distributed and taken in the last five minutes of the presentation and was designed to activate retrieval of newly acquired environmental health information and to determine if the learner outcomes were attained. Data were all multiple choice. (DOCX 25 kb) [file 12909_2018_1266_MOESM3_ESM.docx]

**Additional file 3**

ASSIGNED THREE DIGIT NUMBER:_______

**Environmental Exposure Post-Test:**

1. **Who is more vulnerable to environmental exposure, adults or children?** (circle one)

2. **Choose what is NOT a possible exposure pathway of environmental exposure.**

1. Transplacental
2. Ingestion
3. Inhalation
4. Dermal absorption
5. All of these are potential exposure pathways

3. **Choose 4 health effects of nicotine/secondhand smoke exposure?**

1. Low birth weight
2. Fragile X syndrome
3. Decreased head circumference
4. Ear infections
5. ADHD
6. Cleft palate
7. Autism

4. **Since polychlorinated biphenyls (PCBs) were banned in the US in 1979, are they still an exposure concern for today’s children?** Yes/No (circle one)

5. **Is there a true “safe” level for lead exposure?** Yes/No (circle one)

6. **Each 10-fold increase in DDT level in blood is associated with a 10 to 15 point decrease in mental development.** True/False (circle one)

7. **With long-term exposure, pesticides can affect what body systems?** (choose 4)

1. Endocrine
2. Respiratory
3. Cardiovascular
4. Immune
5. Reproductive
6. Digestive
7. Vision

8. **Where can flame-retardants be found?**

1. Furnishing materials
2. Electronics
3. Plastics
4. Foams
5. All of the above

9. **The “green cleaning” market is highly regulated with many laws and regulations.** True/false (circle one)

10. **What is a major source of BPA (bisphenol A) exposure in children?**

1. Playground equipment
2. Food
3. School supplies (pencils, erasers, etc.)
4. Bubble baths

11. **Children cannot be exposed to air pollution indoors.** True/False (circle one)

12. **Boys are more susceptible** **to behavioral problems associated with increased lead exposure.** Yes/No (circle one)

13. **Are toxic chemicals found in breast milk?** Yes/No (circle one)

14. **Fetuses are protected for environmental toxins *in utero****?*  True/False (circle one)

15. **What brought you to this event today?**

___________________________________________________________________________

___________________________________________________________________________

16. **Do you feel that this information will be helpful to you when you are working in homes? Why or why not?**

___________________________________________________________________________

___________________________________________________________________________

17. **How likely are you to provide information about environmental exposures in the homes of people you visit?**

1(least likely) 2 3 4 5(extremely likely)

18. **Has this event changed your view/understanding of environmental exposures?**

___________________________________________________________________________

___________________________________________________________________________
